# Commanding the Commander: structure of a key protein machinery in endosomal trafficking

**DOI:** 10.1038/s41392-023-01568-4

**Published:** 2023-08-04

**Authors:** Xin Yong, Chunzhuang Zhou, Daniel D. Billadeau, Da Jia

**Affiliations:** 1 Key Laboratory of Birth Defects and Related Diseases of Women and Children, Department of Paediatrics, West China Second University Hospital, State Key Laboratory of Biotherapy, Sichuan University, Chengdu, 610041 China; 2 Division of Oncology Research and Schulze Center for Novel Therapeutics, Mayo Clinic, Rochester, MN 55905 USA

**Keywords:** Structural biology, Cell biology

In a recent study published in *Cell*^1^ *and* two bioRxiv pre-prints,^2,3^ three studies investigate the structure of the endosomal Commander complex through a combination of X-ray crystallography, cryogenic-electron microscopy (Cryo-EM), Alphafold predictions and extensive site-directed mutagenesis (Fig. 1a), which sheds lights on the molecular characteristics of this evolutionarily-conserved protein machinery, and enables the mapping of mutations causing X-linked intellectual disability (XILD) and Ritscher-Schinzel syndrome (RSS).

**Fig. 1:**
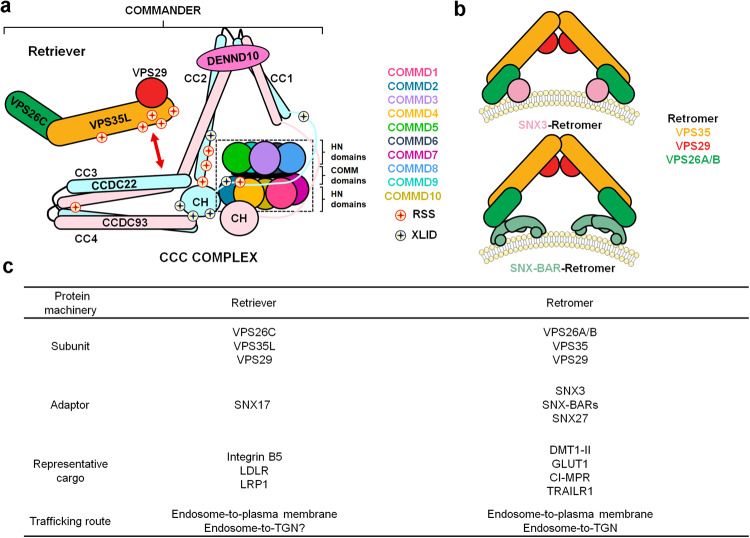
**a** The structure model of the Commander complex (Model Archive: ma-ri7tb), adapted from graphic abstract of the *Cell* study.^1^ **b** The structural models of the SNX3-Retromer (PDB:7BLP) and SNX-BAR-Retromer complex (PDB:6H7W) on the membrane. **c** Comparison of Retriever and Retromer

When proteins enter the endosomal network, they are either transported to lysosome for degradation, or recycled to the plasma membrane or the trans-Golgi network for reuse. Multiple protein machineries are essential for the process, including Retromer, Commander, and several members of the sorting nexin (SNX) family. Commander genes are highly conserved in metazoa and can be found in almost all tissues and cells. The Commander complex is responsible for endosome-to-plasma membrane recycling of a vast array of endosomal proteins (cargos), including integrins and lipoprotein receptors.^4^ These proteins are recognized by Commander adaptor protein SNX17, which specifically binds the NPXY/NXXY motif localized in the cytoplasmic tail of the transmembrane proteins. Commander plays an indispensable role in embryogenesis, and its mutations are linked with XLID and RSS, two rare developmental disorders. Despite its essential role in membrane trafficking and importance in human diseases, the structure and function of Commander remain largely elusive.

The Commander complex consists of sixteen protein subunits. Except for DENND10, the remaining fifteen proteins are organized into two distinct sub-assemblies, known as the Retriever and CCC complexes (Fig. 1a). The Retriever complex comprises three subunits, VPS35L-VPS26C-VPS29, and shares a distant homology to the Retromer complex (VPS35-VPS26A/B-VPS29). The CCC complex consists of a total of twelve components, including two coiled-coil domain-containing proteins (CCDC22 and CCDC93), and ten members of the COMMD (copper metabolism MURR1 domain) family (denoted as COMMD1-10). Notably, the COMMD proteins adopt a unique hetero-decameric closed ring structure, which is primarily maintained through the interplay between the COMMD domains of the ten COMMD proteins (Fig. 1a). The alpha-helical N-terminal (HN) domains of the COMMD proteins are positioned at the periphery of the ring (Fig. 1a).

Both CCDC22 and CCDC93 have an N-terminal calponin-homology (CH) domain and a C-terminal coiled-coil (CC) domain, and a long linker connecting these two domains. Structural analysis and in silico predictions reveal that CCDC22 and CCDC93 are key to the assembly of the Commander complex and interact with the COMMD proteins, DENND10 and Retriever through distinct regions (Fig. 1a). The CH domain of CCDC93 and the long linkers of CCDC22 and CCDC93 are involved in the interactions with the COMMD proteins. Alphafold2 predicts that CCDC22 and CCDC93 to form a heterodimer via the four coiled-coil regions (CC1-CC4), resulting in two V-shaped structures (Fig. 1a). The first V-shape (CC1 and CC2) binds directly to DENND10, forming a stable CCDC22-CCDC93-DENND10 trimer. The second V-shape (CC3 and CC4) is responsible for the association with Retriever via its subunit VPS35L. The association with VPS35L is further stabilized by the CH domain of CCDC22. Remarkably, pathogenic mutations in VPS35L and CCDC22 causing XLID and RSS tend to cluster near the interface between the Retriever and CCC complexes, emphasizing the importance of the correct assembly of the Commander complex (Fig. 1a).

Even though the structural and experimental data demonstrate the subunits of the Commander complex folds into a close-packed structure, the biological functions of its two sub-assemblies are characterized in different levels. In contrast to the CCC complex, the function of Retriever is better understood, in part due to its similarity with Retromer (Fig. 1b and c). These two complexes share a common subunit, VPS29, and the remaining two subunits share a high degree of sequence and structural similarity. Not surprisingly, Retriever and Retromer adopt an analogous architecture, with VPS35L (or VPS35 in Retromer) serving as the main platform for VPS26C (or VPS26A/B in Retromer) and VPS29 binding (Fig. 1a, b). Despite these similarities, Retriever is distinct from Retromer in many different aspects. First, Retromer is known to mediate both endosome-to-plasma membrane and endosome-to-TGN trafficking, whereas Retriever is reported to be only involved in the endosome-to-plasma membrane protein trafficking for now (Fig. 1c). Second, Retromer associates multiple cargo adaptors, such as SNX3, SNX27 and SNX-BARs, and recognizes and transports cargos with various motifs, such as Øx(L/M/V), PDZbm or SNX-BAR binding motifs.^5^ In contrast, Retriever recognizes and recycles cargoes harboring NPXY/NXXY motif via its adaptor protein SNX17 (Fig. 1c). However, additional analysis is required to determine whether other adaptors and cargoes are involved in the Retriever or Commander pathway. Third, Retriever is more compact than Retromer, and harbors many unique residues that are critical for contacting the CCC complex. Last but not the least, VPS29 has a solvent exposed surface in the Retromer structure, and many Retromer-associated proteins, such as TBC1D5 and VARP, interact with Retromer through this interface. In the Retriever structure, the VPS29 interface engages with a peptide from the N-terminus of VPS35, preventing the interaction with Retromer-associated proteins.

Whereas the results of the two pre-prints are mostly in accordance with the *Cell* paper, Laulumaa et al.^2^ utilized cross-linking mass spectrometry (XL-MS) and cryo-EM to obtain a detailed structure of the Commander complex and discovered numerous new binding partners of Commander, thus bringing new perspectives into the functions of the Commander complex. Furthermore, Boesch et al.^3^ presented a higher-resolution structure of Retriever (2.9 Å), which allowed them to precisely determine the impact of cancer-related mutations in Commander.

To summarize, three studies collectively provide a molecular rationale for the assembly of the Commander complex and represents an important step in understanding the molecular mechanisms regulating different endocytic trafficking pathways. However, many important questions remain to be understood. First, what is the function of Commander, other than regulating endosomal trafficking of SNX17 cargoes? In part, we know that the CCC subcomplex regulates PI(3)P levels on endosomes through the recruitment of MTMR2, a PI(3)P phosphatase.^6^ Loss of the CCC subcomplex leads to enhanced endosomal PI(3)P resulting in the hyper-recruitment and activation of the Arp2/3-activating WASH complex. Second, how do Commander and SNX17 coordinate to mediate the protein trafficking? Third, how does Commander assemble on the endosomal membrane? Previous data suggests that Commander recruitment relies on an interaction of CCDC93 with the WASH complex subunit FAM21. Whether this is the only interaction that is required or if other protein-protein or protein-phospholipid interactions are involved remain to be determined. Interestingly, COMMD proteins have been shown to interact with phospholipids,^7^ thus one could envision an additional unique mechanism of Commander recruitment through the COMMD dodecamer with certain phosphatidylinositol-enriched membranes.

## Data Availability

Data supporting the findings of this study are available within the paper.
